# Sustained effort network for treatment of status epilepticus/European academy of neurology registry on adult refractory status epilepticus (SENSE-II/AROUSE)

**DOI:** 10.1186/s12883-023-03505-y

**Published:** 2024-01-04

**Authors:** Charlotte Damien, Markus Leitinger, Christoph Kellinghaus, Adam Strzelczyk, Pia De Stefano, Christoph P. Beier, Raoul Sutter, Leena Kämppi, Daniel Strbian, Erik Taubøll, Felix Rosenow, Raimund Helbok, Stephan Rüegg, Maxwell Damian, Eugen Trinka, Nicolas Gaspard

**Affiliations:** 1 Department of Neurology, Hôpital Universitaire de Bruxelles, Hôpital Erasme, Brussels, Belgium; 2 Department of Neurology Neurointensive Care and Neurorehabilitation, Centre for Cognitive Neuroscience, Christian Doppler University Hospital, Paracelsus Medical University, European Reference Network EpiCARE, Salzburg, Austria; 3 Neuroscience Institute, Department of Neurology, Centre for Cognitive Neuroscience, Christian Doppler University Hospital, Paracelsus Medical University, Salzburg, Austria; 4 Epilepsy Center Münster-Osnabrück, Klinikum Osnabrück, Osnabrück, Germany; 5 Department of Neurology and Epilepsy Center Frankfurt Rhine-Main, Goethe-University and University Hospital Frankfurt, Frankfurt am Main, Germany; 6 EEG & Epilepsy Unit, Department of Clinical Neurosciences, University Hospital of Geneva, Geneva, Switzerland; 7 Neuro-Intensive Care Unit, Department of Intensive Care, University Hospital of Geneva, Geneva, Switzerland; 8 Department of Neurology, Odense University Hospital, Odense, Denmark; 9 Department of Neurology, University Hospital Basel, Basel, Switzerland; 10 Intensive Care Units, University Hospital Basel, Basel, Switzerland; 11 Department of Neurology, Epilepsia Helsinki, Helsinki University Hospital and University of Helsinki, Helsinki, Finland; 12 Department of Neurology, Oslo University Hospital, Oslo, Norway; 13 Department of Neurology, Johannes Kepler University Linz, Linz, Austria; 14 Department of Critical Care, Essex Cardiothoracic Centre, Basildon, UK; 15 Karl Landsteiner Institute of Neurorehabilitation and Space Neurology, Salzburg, Austria; 16 Department of Public Health, Health Services Research and Health Technology Assessment, UMIT – University for Health Sciences, Medical Informatics and Technology, Hall en Tyrol, Austria; 17 Department of Neurology, Yale University School of Medicine, New Haven, CT USA

**Keywords:** Status Epilepticus, Outcome, Refractoriness, Anti-seizure medication, Continuous intravenous anesthetic Drugs, EEG

## Abstract

**Background:**

Status Epilepticus (SE) is a common neurological emergency associated with a high rate of functional decline and mortality. Large randomized trials have addressed the early phases of treatment for convulsive SE. However, evidence regarding third-line anesthetic treatment and the treatment of nonconvulsive status epilepticus (NCSE) is scarce. One trial addressing management of refractory SE with deep general anesthesia was terminated early due to insufficient recruitment. Multicenter prospective registries, including the Sustained Effort Network for treatment of Status Epilepticus (SENSE), have shed some light on these questions, but many answers are still lacking, such as the influence exerted by distinct EEG patterns in NCSE on the outcome. We therefore initiated a new prospective multicenter observational registry to collect clinical and EEG data that combined may further help in clinical decision-making and defining SE.

**Methods:**

Sustained effort network for treatment of status epilepticus/European Academy of Neurology Registry on refractory Status Epilepticus (SENSE-II/AROUSE) is a prospective, multicenter registry for patients treated for SE. The primary objectives are to document patient and SE characteristics, treatment modalities, EEG, neuroimaging data, and outcome of consecutive adults admitted for SE treatment in each of the participating centers and to identify factors associated with outcome and refractoriness. To reach sufficient statistical power for multivariate analysis, a cohort size of 3000 patients is targeted.

**Discussion:**

The data collected for the registry will provide both valuable EEG data and information about specific treatment steps in different patient groups with SE. Eventually, the data will support clinical decision-making and may further guide the planning of clinical trials. Finally, it could help to redefine NCSE and its management.

**Trial registration:**

NCT number: NCT05839418.

**Supplementary Information:**

The online version contains supplementary material available at 10.1186/s12883-023-03505-y.

## Background

Status epilepticus (SE) is a common neurological emergency defined by the International League Against Epilepsy (ILAE) Task Force on Classification of SE as “ a condition resulting either from the failure of the mechanisms responsible for seizure termination or from the initiation of mechanisms which lead to abnormally prolonged seizures and can have long-term consequences, including neuronal injury or death, and alteration of neuronal networks, depending on the type and duration of seizures” [1]. The duration threshold has been defined for convulsive SE as a seizure lasting longer than 5 min, for focal and/or non-convulsive SE as lasting longer than 10 min or for non-convulsive SE for a total duration of 20% of any 60-minutes period of recording [2, 3].

The current treatment strategies of SE use a tiered approach, in which the sequential administration of different drugs follow pre-defined temporal points. These may not always reflect the severity of the SE episode and need for urgent seizure cessation, hence are rarely strictly adhered to in clinical practice [4–6]. In early SE (5 to 20 min after seizure onset), a parenteral benzodiazepine should be given. In established SE (20 to 40 min) a non-sedating intravenous (IV) anti-seizure medication (ASM) is recommended. Refractory status epilepticus (RSE; 40 to 60 min) is defined as SE persisting despite administration of at least two appropriately selected and dosed parenteral medications, including a benzodiazepine. Super-refractory SE (SRSE) is defined as SE persisting or recurring after 24 h or more of treatment with continuous intravenous anesthetic drugs (CIVADs) [7].

In the last three decades, a few large randomized controlled trials provided evidence for the early stages of SE and established the role of benzodiazepines [8–10]. However, underdosing and/or absence of benzodiazepines as first-line treatment have been widely observed and has been associated with poorer outcome, among others things [11–14].

In 2019, the multicenter randomized double-blind “Established Status Epilepticus Treatment Trial” (ESETT) compared IV fosphenytoin, levetiracetam and valproate for established convulsive status epilepticus (CSE), with similar rates of SE cessation and adverse events, confirmed by recent pediatric studies [15]. Based on these trials, the first and second-line treatments of convulsive SE are well established. Previous studies also suggested that other non-sedative IV ASMs, such as lacosamide or brivaracetam could be effective and safe as second-line treatment or beyond [16–23, 23, 24].

Although CIVADs are recommended as third-line treatment and are safe to use as second-line [25], there is currently limited evidence to guide this third-line and later treatments on convulsive and other types of SE.

In RSE, a randomized trial comparing propofol and barbiturates was initiated in 2006 but was terminated early due to insufficient enrollment. Efficacy was similar between the two drugs but duration of intubation was much longer with barbiturates [26]. Few observational studies compared anesthetic drugs and different doses [27–29]. Overall, data support the choice of midazolam and propofol to treat refractory SE over barbiturates, which are associated with significantly more complications (especially infections) and mortality, but randomized controlled trials are lacking [30–32]. Non-sedating ASMs are an alternative to CIVADs in refractory nonconvulsive cases, but there is a lack of evidence regarding which drug to administer and how, as highlighted by several recent reviews [33] and observational case series [34–36].

There are even fewer data to guide the management of super-refractory SE [37] and for the use of uncommon strategies in this setting, including ketamine, isoflurane [38] the ketogenic diet, electroconvulsive therapy, and neurostimulation [39, 40], hypothermia or immune therapies in SE of suspected autoimmune or autoinflammatory etiology [41, 42].

Further investigation is needed to determine the optimal EEG target in patients with RSE and SRSE. Commonly used EEG surrogates of RSE control include seizure suppression, the emergence of a burst-suppression pattern (BS), or an isoelectric background. Achieving these surrogate endpoints often requires titrating intravenous antiepileptic drugs based on EEG readings. A multinational survey favored BS as the preferred target for CIVAD titration in RSE patients [43], with an interburst interval of around 10 s suggested by experts [44]. Achieving BS could be challenging and was not associated with sustained seizure termination or better outcome in a recent observational study [45]. The adequacy of BS as a surrogate target then lacks substantial evidence [46].

Finally, although the treatment of CSE is well established, these recommendations are often extrapolated to NCSE due to lack of data confirming the appropriateness of this practice. Currently, there is no clear consensus on the treatment of NCSE.

To address all these issues, randomized and controlled trials are needed. Such trials require substantial financial and human resources to achieve sufficient statistical power [47]. This is a challenge for RSE and SRSE, which have a low annual incidence of respectively up to 7 per 100.000 adults and 1 per 100.000 adults [48–50]. Other limitations include the challenges of clinical trials in the emergency setting and in temporarily incapacitated subjects.

Multicenter prospective observational registries offer an alternative approach to randomized controlled trials by collecting data on determinants of outcome, practice variability and allow comparative effectiveness and efficacy studies. Recently, the Sustained Effort Network for treatment of Status Epilepticus (SENSE) has provided real-life data about SE [51–55]. Between January 2011 and June 2015, 1179 episodes of SE in 1049 subjects from eight German-speaking centers in Austria, Germany, and Switzerland, were enrolled in SENSE. Collected data helped provide new insights into the factors contributing to SE cessation and refractoriness, functional outcome, and intubation.

The overarching goal of the Sustained Effort Network for Treatment of Status Epilepticus/European Academy of Neurology Registry on Refractory Status Epilepticus (SENSE-II/AROUSE) is to harmonize the collection of clinical and EEG data from multiple centers on SE to gain further insight on the management of SE across all its semiologies and etiologies, and to compare second- and third-line treatments and beyond. More specifically, we aim to:

- identify risk factors of outcome, refractoriness, super-refractoriness.
- perform comparative effectiveness studies on third-line treatment and beyond, and on the use of cEEG.
- collect epidemiological data on rare forms and causes of SE.
- identify key targets for future randomized controlled trials.

## Methods/Design

### Study design

SENSE-II/AROUSE is a prospective, multicenter, observational registry study on consecutive cases of SE in patients 18 years of age or older. We aim to involve university hospitals as well as non-university hospitals to enhance generalizability of our findings.

Currently, 12 high-volume medical centers in Belgium, Austria, Germany, Switzerland, Norway, Finland, and Denmark are involved:

- Belgium : Hôpital Universitaire de Bruxelles – Erasme Hospital (C. Damien, N. Gaspard) ; Centre Hospitalier Universitaire Brugmann (C.Damien).
- Austria: University Hospital Salzburg - Christian Doppler University Hospital (M. Leitinger, E.Trinka) ; Johannes Kepler University Linz (R. Helbok).
- Germany: Goethe-Universität Frankfurt am Main (A. Strzelczyk, F. Rosenow); Klinikum Osnabrück (C. Kellinghaus).
- United Kingdom: University Hospitals Birmingham (M. Damian).
- Switzerland: Universitätsspital Basel (S. Rüegg, R. Sutter); University Hospital of Geneva (P. De Stefano).
- Norway: Oslo University Hospital (E. Taubøll).
- Finland: HUS Helsinki University Hospital (L. Kämppi).
- Denmark: Odense University Hospital (C. Beier).

Personnel collecting data from the patient charts and entering data into the database are board certified neurologists, or neurology residents, or medical students in their final year, or study nurses, who will be specifically instructed prior to data collection and personally supervised by one of the principal investigators during the data collection and entry.

Patients will be treated in emergency departments, EEG-laboratories, or intensive care units with electronical data entry and management, or with dedicated data collection sheets in place [56]; thus, type, dosages and time points of drug administration will be easily ascertained. The increasing use of continuous video-EEG (cEEG) monitoring will also allow for exact and reliable determination of cessation of NCSE, and in some cases also of NCSE onset.

### Objectives

The main objectives of the study are to assess patient characteristics, treatment modalities, to analyze and collect EEG features and outcome of adults treated for SE and identify predictors of outcome. Data assessed in this study could also identify gaps and opportunities of the management of this medical emergency.

More specifically, the following questions will be addressed:

1. Which factors determine global outcome at the time of hospital discharge?
2. Which factors are associated with refractoriness and super-refractoriness?
3. What are the efficacy and safety issues associated with each treatment?
4. What are the characteristics of EEG in NCSE and do they carry clinically relevant information?
5. How could we better classify SE taking semiology, etiology, and EEG into account?
6. Are there differences in the pattern of care and outcome between the centers?

### Study population

We aim to enter all consecutive adult patients who are diagnosed with SE at admission or at any point of the inpatient treatment into the registry.

SE is defined as:

- Convulsive, i.e., bilateral tonic-clonic, seizure lasting 5 min or longer [1].
- SE with prominent motor symptoms other than convulsive SE lasting 5 or more minutes [1].
- Clinical seizures without prominent motor manifestations lasting 10 min or longer [1, 2].
- Recurrent seizures without regaining neurological baseline status between them and altogether fulfilling the above mentioned diagnostic time criteria [1, 57].
- Nonconvulsive or possible electrographic SE (synonymous: ictal-interictal continuum, IIC) diagnosed with EEG according to Salzburg Criteria [58, 59] or American Clinical Neurophysiology Society (ACNS) terminology 2021 (Electrographic SE is defined as an electrographic for ≥ 10 continuous minutes or for a total duration of > 20% of any 60-minute period of recording) [2].

RSE is defined as SE persisting despite administration of at least two appropriately selected and dosed parenteral medications including a benzodiazepine. SRSE is defined as SE persisting or recurring after 24 h or more of treatment with CIVADs [6].

### Inclusion and exclusion criteria

Post-anoxic SE is considered to be distinct from other forms of SE and carries a very specific and generally poor prognosis. Therefore, we will exclude patients with post-anoxic SE. No other exclusion criteria will apply besides age (< 18 years).

### Clinical data

Variables in Tables 1, 2 and 3 will be collected.

**Table 1:** Baseline, SE-related and outcome variables; SE: Status Epilepticus; mRS: modified Rankin Scale; ILAE: International League Against Epilepsy; NCSE: non convulsive status epilepticus; EMSE score: Epidemiology Based Mortality Score in Status Epilepticus

**Demographics and premorbid status**	Age at admission, gender, ethnicity Estimated weight on admission. Location at SE onset Date and time of hospital admission Premorbid Clinical Frailty Scale [60] and premorbid mRS [61]
**Health variables**	History of previous seizures/epilepsy, history of SE Relevant known health problems (comorbidities) as found in Charlson comorbidities index [62]
**SE-related variables** General	Date and time of SE onset Refractory and super-refractory SE Date and time of refractoriness and super refractoriness
Semiology	Semiology according the ILAE [1] Worst seizure type according to STESS score [63] Level of consciousness at SE diagnosis or onset
Etiology	Etiology according to the ILAE and according to the EMSE score SE episode fulfilling criteria of New Onset Refractory Status Epilepticus (NORSE) or Febrile Infection-Related Epilepsy Syndrome (FIRES) [7]
**EEG-related variables**	Date of ictal EEG (if applicable) EEG pattern *(according to the ACNS 2021 terminology and Salzburg criteria for NCSE)* [2, 58, 59] EEG findings as classified in EMSE score [50] If NCSE was diagnosed by EEG and clinical response to IV ASM, then the ASMs and the timing and kind of EEG and clinical improvement are documented. Use of cEEG or repeated EEG in management/diagnosis Used EEG-target for CIVAD : seizure suppression, burst-suppression or flat EEG
**Performed diagnosis procedures**	Brain CT or MRI, continuous video-EEG monitoring (cEEG), fluorine-18 fluorodeoxyglucose (FG) brain positron emission tomography (PET) scan, cerebrospinal fluid analysis, antineuronal antibodies research, brain biopsy, therapeutic drug monitoring, Multimodal Invasive Monitoring or other.
**Outcome-related variables**	Date and time of SE cessation and EEG-documentation of SE cessation Date and time of first-time following commands Date of discharge from hospital mRS at discharge and at 30 days after admission (if still hospitalized) At 90 days after discharge: mRS, recurrence of seizure, ongoing ASM therapy Date and cause of death Reason for withdrawal of life support therapies

**Table 2:** Treatment-related variables; CIVAD: continuous intravenous anaesthetic drug; EEG: electroencephalography; IV: intravenous; ASM: antiseizure medication; NCSE: nonconvulsive status epilepticus

**First-line Benzodiazepines**	Use of benzodiazepines as first-line treatment Location, date and time of administration Bolus dose and mode of administration
**Second-line and other uses of conventional ASMs and benzodiazepines** (beyond first-line)	Date and time of first administration of any ASM used (including benzodiazepines and conventional ASMs) Bolus dose and mode of administration Maximum daily dose and mode of administration Date and time of last dose applied and reason for discarding the substance. Maximum serum levels of the substances in use (if applicable) Adverse event documentation (if applicable)
**Diagnostic IV ASM trial in IIC**	If NCSE was diagnosed by EEG and clinical response to IV ASM, then the ASMs and the timing and kind of EEG and clinical improvement are documented.
**CIVADs**	Date and time of initiation of any CIVADs Bolus dose, initial and maximal infusion rate Date and time of discontinuation and reason for discontinuation Adverse event documentation (if applicable)
**Inhalational drugs**	Date and time of initiation of any inhalational drugs System of delivery Initial and maximal inhalation rate Date and time of discontinuation and reason for discontinuation.
**Immune therapies**	Use of immune therapy: steroid, intravenous immunoglobulins, plasma exchange, immune-adsorption, cyclophosphamide, rituximab, anakinra, tocilizumab or others. Date and time of initiation and of discontinuation Maintenance dosage and total amount applied
**Additional therapies**	Use of additional therapy: ketogenic diet, vagus nerve stimulation, hypothermia, electroconvulsive therapy and other neurostimulations, neurosurgical procedure or other Date and time of initiation and of discontinuation.

**Table 3:** Complications and life support therapies-related variable; ICU: intensive care unit; SE: status epilepticus

**Intubation**	Date and time (if applicable) Reason for intubation (escalation of SE treatment vs. other reason) Duration of intubation
**Vasopressors**	Date and time of initiation of any vasopressor (if applicable) Duration of vasopressors need (hours)
**ICU stay**	Need for ICU stay and duration of ICU stay
**Other**	Other organ support requirement (renal replacement therapies, etc.)

The modified Rankin Scale (mRS) [61] –a global outcome measure of morbidity and impairment -was chosen because of its easy applicability in a large variety of clinical scenarios with neurological diseases.

The premorbid Clinical Frailty Scale [60] was chosen in addition to the mRS for its greater sensitivity to premorbid functional status.

Of note, this study does not require any additional investigation apart from standard treatment and does not interfere in any way with the treatment decisions made by physicians during the inpatient phase. However, the patient will be called 90 days after onset/ discharge, which requires informed consent obtained during the inpatient phase in some countries.

### Management protocol

No common management protocol will be imposed on the participating centers. However, most institutions have established a local protocol that is closely related to the most recent guidelines for the management of SE published by the American Epilepsy Society (AES) in 2016 [64].

In general, diagnosis of SE and related conditions is made based on clinical suspicion using physical examination and, where necessary, EEG (including, cEEG).

In most cases, emergency cerebral imaging with CT or MRI as well as standard emergency blood analysis, and CSF analysis if indicated, will be performed. Most frequently, the first treatment step consists of the administration of a benzodiazepine. If unsuccessful, intravenous administration of ASMs such as levetiracetam, valproate, (fos-)phenytoin, or lacosamide follows. As a third step, another ASM is added or anesthetics such as propofol or midazolam. All treatment decisions will remain at the discretion of the attending physicians.

The use of data acquisition sheets for the very early stages SE (Annex; modified from [56]) and ASM trials for IIC, which are already in place in some centers, will be encouraged to facilitate homogenous and complete data collection.

All participating centers perform cEEG for the following indications:

- Management of refractory and super-refractory SE.
- Suspicion of NCSE after a convulsive SE or seizure (lack of return to neurological baseline).

Some participating perform cEEG for additional indications, such as patients unexplained altered mental status, detection of delayed cerebral ischemia in aneurysmal, subarachnoid hemorrhage.

The availability of cEEG is specific to each center, varying from 7 days a week (including Sundays and holidays) to during working hours on weekdays.

### Data security and quality assessment

Recorded data are pseudonymized using site-specific sequential alphanumeric codes known to the local investigators only. Data entry is compliant with General Data Protection Regulation (GDPR) 2016/679 of European Union law. In countries where more restrict data protection rules are in place, only anonymized data will be provided.

Data collection will be performed using Research Electronic Data Capture (REDCap) electronic data capture tools hosted at Erasme Hospital [65, 66] REDCap is a secure, web-based software platform designed to support data capture for research studies, providing (1) an intuitive interface for validated data capture, (2) audit trails for tracking data manipulation and export procedures, (3) automated export procedures for seamless data downloads to common statistical packages, and (4) procedures for data integration and interoperability with external sources. Data are collected prospectively from the admission of the patient to discharge. Documentation must be started latest at the third working day after treatment to guarantee data quality. In some centers, data collection will be not feasible for a limited number of weeks for organizational reasons. These times will be communicated in advance to the steering committee. However, in the other time the consecutive inclusion of patients will be guaranteed.

Center participation will be monitored by a study coordinator to ensure consecutive case assessment and timely data entry. An independent data monitoring committee will periodically review data entry for completeness and accuracy. Any query due to missing data or lack of consistency will be resolved with local investigators.

### Ethics approval

The local ethics committee of each participating center approved the final study protocol. The following ethics committees reviewed and approved the study.

The study was registered at the Ethics Committee of Erasme Hospital (Committee Reference Number: 406; Protocol Number: CCB: B4062020000170; P2020/483).

The study was also registered in PRS with number NCT05839418.

In Switzerland, the collected data being part of this registry will not be used for research purpose (analyzed or published) without the formal approval by the Regional Research Ethic Committee (Ethikkommission Nordwest- und Zentralschweiz for Basel -EKNZ- and Commission Cantonale d’Ethique de la Recherche sur l’être humain for Geneva -CCER-).

This study will be performed in line with the principles of the Declaration of Helsinki.

### Informed consent

In some countries, informed consent was waived due to the non-interventional character of this study. In other countries, informed consent is obtained as early as possible once the patient recovers from SE. If a patient does not recover and a legal representative is available, then this legal representative will provide informed consent. This rule also applies if a legal representative or a health care proxy has already been in place before SE occurred. If the patient dies, data are included for ethical reasons in terms of safety considerations. Informed consent is particularly necessary for the telephone call 90 days after discharge in some countries. If no informed consent can be obtained, the patient will only be logged as event but medical data will be deleted in some countries.

In some countries, informed consent is necessary purely due to the prospective nature of this study and due to data sharing regulations, strictly adhering to the local regulations regarding secondary usage of data collected for the routine clinical care.

### Sample size

In the first prospective registry SENSE [51, 52], refractoriness rate reached 50%. Recent retrospective studies, single-center or population-based, showed a super-refractoriness rate from 3 to 10% [49, 67] using criteria defined above.

Thus, a cohort of approximately 3000 patients will be needed to reach a group size of 1500 patients in the refractory group and of about 150 patients in the super-refractory group.

For the refractory group, this group size is necessary to assess over 50 possible factors associated with outcome with a two-sided significance level of 5%, a power of 80% and an anticipated effect size of 10%.

For the super-refractory group, this group size will be needed to assess approximately 15 possible predictors of outcome with a two-sided significance level of 5%, a power of 80% and an anticipated effect size of 10%.

### Data analysis

Calculations will be conducted according to incident patients (especially for mortality, where each patient cannot count for several exposures), and episodes (for example, regarding treatment impact, where several exposures of the same patient are potentially informative).

Continuous variables will be presented as mean and standard deviation in case of a normal distribution and as median and interquartile range with variables without normal distribution.

Interval-scaled and ordinal-scaled variables will be presented as median and interquartile range.

Categorical variables will be presented as frequency and percentages.

For continuous variables, comparisons between outcome categories will be analyzed using parametric tests for normally distributed variables and non-parametric tests for non-normally distributed variables. For categorical variables, comparisons will be performed using the Pearson’s Chi-squared test or Fisher’s exact test. Multivariable approaches will assess significance of variables of interest after multivariable analyses. Statistical analyses will be performed using the latest version of R (R Core Team).

## Discussion

SENSE allowed to include over 1000 patients with SE from 8 centers. The study provided real-life data about SE and in particular refractory SE [54, 55].

It confirmed that a large proportion of first-line treatments was underdosed compared to the guidelines [54]. First-line treatment did not include benzodiazepines in 20% of all cases, 6% and 22% in CSE and non-CSE, respectively. These findings strongly suggest that IV ASMs are less efficacious than benzodiazepines as first-line treatment. Several predictors of SE cessation have been identified, such as a younger age, a lower mRS before SE onset, lower number of comorbidities, benzodiazepine as first-line treatment and a higher cumulative dose of ASMs within the first 30 min [53, 54]. Refractoriness occurred in 55% of patients and was associated with a higher baseline mRS and treatment deviation from guidelines, such as a lower bolus dose than recommended [54]. Unlike other studies, no difference was found comparing etiologies of refractory and non-refractory SE. Variables associated with good outcome in RSE were lower SE Severity Score (STESS) at SE onset, a shorter SE duration, a shorter length of stay and a lack of intubation. More than 70% of refractory SE were treated without airways protection and or mechanical ventilation. Limitations mentioned by the authors were the variability in recruitment of patients through the different centers, who could decrease the cohort homogeneity and potentially impacting on generalizability. Continuous EEGs were not available for most patients, even though it was recommended by several scientific societies [64, 68]. The focus of SENSE was RSE but only a few SRSE were recruited.

SENSE-II/AROUSE will provide high-quality data on SE of all types and severities. We have planned to collect continuous EEG data, if applicable, to alleviate the lack of knowledge about electrographic patterns of NCSE. We will focus on the functional outcome of SE but also on the refractoriness and super-refractoriness to identify potential predictors. Finally, due to lack of studies of third-line treatment and beyond, we plan to perform a comparative effectiveness study based on the collected data.

The evaluation of a large, heterogeneous patient group will hopefully help in clinical decision making in an area where data with higher level of evidence are still lacking.

### Electronic supplementary material

Below is the link to the electronic supplementary material.

Supplementary Material 1

## Data Availability

Not applicable.

## Abbreviations

ACNS: American Clinical Neurophysiological Society
AES: American Epilepsy Society
ASM: Anti-seizure medication
CIVADs: Continuous intravenous anesthetic drugs
CSE: Convulsive status epilepticus (i.e., bilateral tonic clonic)
CT: Computer tomography
EEG: Electroencephalogram
EMSE: Epidemiology-based mortality score in status epilepticus
ESETT: Established status epilepticus treatment trial
FDG: Fluorine-18 fluorodeoxyglucose
GDPR: General Data Protection Regulation
IC: Informed consent
ICC: Ictal-interictal continuum
ICU: Intensive care units
ILAE: International League Against Epilepsy
IV: Intravenous
MRI: Magnetic resonance imaging
mRS: Modified Rankin Scale
NCSE: Nonconvulsive status epilepticus
NORSE: New Onset Refractory Status Epilepticus
PET: Positron emission tomography
REDCap: Research Electronic Data Capture
RSE: Refractory status epilepticus
SE: Status epilepticus
SENSE: Sustained effort network for treatment of status epilepticus
SENSE-II/AROUSE: Sustained effort network for treatment of status epilepticus/European Academy of Neurology Registry on refractory Status Epilepticus
SRSE: Super refractory status epilepticus
STESS: Status epilepticus severity score
