# Supplementary figures and images for: Sustained effort network for treatment of status epilepticus/European academy of neurology registry on adult refractory status epilepticus (SENSE-II/AROUSE)
Source: BMC Neurol. 2024 Jan 4;24:19. doi: 10.1186/s12883-023-03505-y (PMC10765797; doi:10.1186/s12883-023-03505-y)

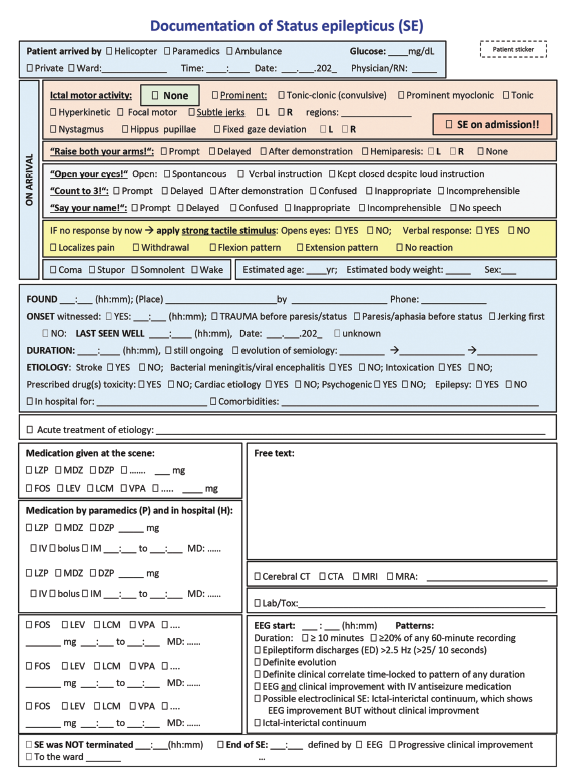

Supplement: Supplementary file 1 — Supplementary Material 1 [file 12883_2023_3505_MOESM1_ESM.png]
